# Long-Term *In Vitro* System for Maintenance and Amplification of Root-Knot Nematodes in *Cucumis sativus* Roots

**DOI:** 10.3389/fpls.2016.00124

**Published:** 2016-02-22

**Authors:** Fernando E. Díaz-Manzano, Rocío Olmo, Javier Cabrera, Marta Barcala, Carolina Escobar, Carmen Fenoll

**Affiliations:** Área de Fisiología Vegetal, Facultad de Ciencias Ambientales y Bioquímica, Universidad de Castilla-La ManchaToledo, Spain

**Keywords:** amplification, *Meloidogyne* sp., root-knot nematodes, cucumber, *in vitro* culture, monoxenic

## Abstract

Root-knot nematodes (RKN) are polyphagous plant-parasitic roundworms that produce large crop losses, representing a relevant agricultural pest worldwide. After infection, they induce swollen root structures called galls containing giant cells (GCs) indispensable for nematode development. Among efficient control methods are biotechnology-based strategies that require a deep knowledge of underlying molecular processes during the plant-nematode interaction. Methods of achieving this knowledge include the application of molecular biology techniques such as transcriptomics (as massive sequencing or microarray hybridization), proteomics or metabolomics. These require aseptic experimental conditions, as undetected contamination with other microorganisms could compromise the interpretation of the results. Herein, we present a simple, efficient and long-term method for nematode amplification on cucumber roots grown *in vitro*. Amplification of juveniles (J2) from the starting inoculum is around 40-fold. The method was validated for three *Meloidogyne* species (*Meloidogyne javanica*, *M. incognita*, and *M. arenaria*), producing viable and robust freshly hatched J2s. These J2s can be used for further *in vitro* infection of different plant species such as *Arabidopsis*, tobacco and tomato, as well as to maintain and amplify the population. The method allowed maintenance of around 90 *Meloidogyne* sp. generations (one every 2 months) from a single initial female over 15 years.

## Introduction

Root-knot nematodes (RKNs; *Meloidogyne* sp.) constitute major pests in agriculture worldwide, causing annual economic losses estimated at $118 billion, (McCarter, 2008). They are obligate parasites that penetrate plant roots to establish their feeding sites, called giant cells (GCs), causing thickenings or knots in the roots referred to as galls (Escobar et al., 2015). *Meloidogyne javanica* (*Mj*), *M. incognita* (*Mi*), and *M. arenaria* (*Ma*) are the most common species of RKNs in the warm climate of southern Europe but also in glasshouses of the more temperate climate of northern Europe (Wesemael et al., 2011). The polyphagous behavior of RKNs as well as the ban on the most effective agrochemical nematicides constitutes a challenge for the successful management of this pest (Haydock et al., 2013). Understanding the molecular processes underlying the formation of galls and GCs and a deep knowledge of the nematode’s biology are crucial for the development of new biotechnology-based control methods (reviewed in Fosu-Nyarko and Jones, 2015). Efficient nematode amplification and maintenance in the laboratory under aseptic experimental conditions are important and quite valuable for molecular biology techniques, such as transcriptomics (as massive sequencing or microarray hybridization), proteomics, metabolomics, etc., being free of biological contamination. So far, the usual practice is to surface-sterilize either eggs or nematode juveniles (J2s) from greenhouse-grown host plant specimens. Normally, a combination of different disinfection methods (mercuric chloride, chlorhexidine, streptomycin sulfate, bleach, antibiotics, physical filters, etc.; Huettel, 1990) is used; however, excessive doses of disinfectants may be toxic, leading to poor nematode survival, whereas insufficient dosage will not ensure efficient sterilization. *In vitro* culture presents the additional advantages of a reduced growth chamber space and non-daily maintenance. Therefore, several protocols for monoxenic nematode cultures have been developed: for example, in tomato excised roots or seedlings (Sayre, 1958; Mathur et al., 1980; Orion et al., 1980; Sudirman and Webster, 1995; Hutangura et al., 1998); in *Abelmoschus esculentus* (Tanda et al., 1980); in onion root cultures (Mitkowski and Abawi, 2002); in *Agrobacterium rhizogenes*-transformed roots of potato, tomato, bindweed, tropical tomato, lima bean, and carrot (Verdejo et al., 1988; Mitkowski and Abawi, 2002). Hydroponic or semi-hydroponic cultures have also been described (Atamian et al., 2012) but nematodes and plants were not completely aseptic.

Here, we describe a simple monoxenic culture method using cucumber roots (*Cucumis sativus*) to amplify different *Meloidogyne* spp. populations. This efficient method provides a viable and prolonged culture system for these obligate plant parasitic nematodes. The main proof of concept is that a population of *M. javanica* has been established from a single female and has been maintained in our laboratory for more than 15 years. The amplification has been successful at a ratio around 40 Pf/Pi (final population/initial population) from the initial J2s inoculum for each re-inoculation obtained. Moreover, J2s from these cultures can be used directly after a simple hatching step in aseptic conditions to infect plants for experiments with different *in vitro* grown plant species. Successful experiments have been performed with this procedure on different genotypes of *Arabidopsis*, tobacco, and tomato (Barcala et al., 2010; Escobar et al., 2010; Portillo et al., 2013; Cabrera et al., 2014, 2015, 2016).

## Materials and Methods

**FIGURE 1:**
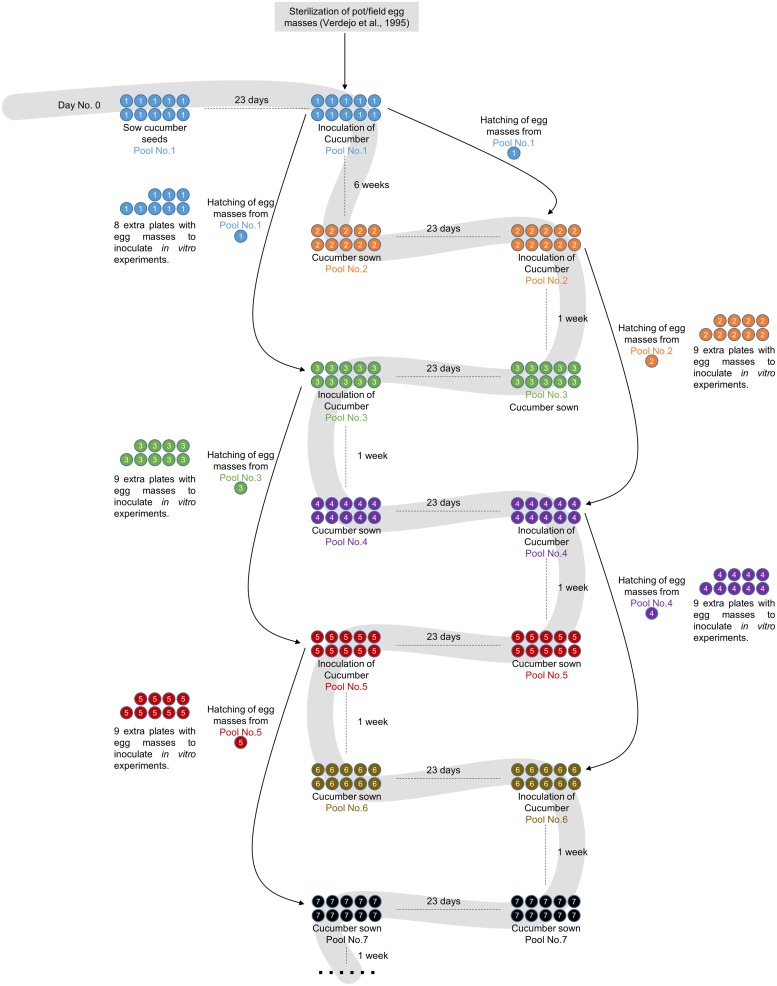
**Flowchart showing the root-knot nematodes (RKN) maintenance and amplification protocol.** Pool No. 1 consists of 10 cucumber plates inoculated with sterilized egg masses from soil-grown plants as described in Verdejo-Lucas (1995). The plates are incubated for 2 months to obtain egg masses. The population of nematodes is maintained in successive cucumber pools grown for 3 weeks after 2 days of stratification at 4°C before inoculation. The nematodes for inoculation are always obtained by hatching egg masses from plates 2 months after inoculation. As only one plate is needed for each amplification round, nine extra plates are always left in order to provide nematodes for *in vitro* experiments or to safeguard the population in the case of contamination or other problems.

The monoxenic nematode culture on cucumber roots was initiated 15 years ago, when egg masses from soil-grown infected tomato plants were used (**Figure 1**). These egg masses were used to obtain sterilized eggs according to Verdejo-Lucas (1995) to inoculate the first pool of 21-day-old cucumber seedlings grown *in vitro*. Every month a new cucumber batch was inoculated with the J2s hatched from egg masses produced in cucumber roots that were inoculated 2 months before (**Figure 1**). A step-by-step protocol with references to all commercial products, materials and tips is provided as Supplemental Material.

### Plant Materials and Growth Conditions

**FIGURE 2:**
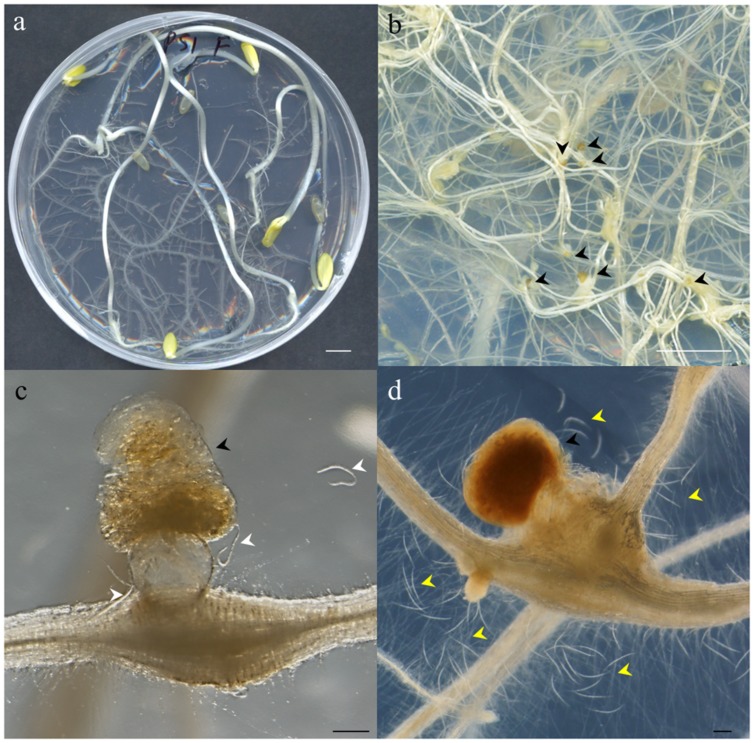
**Photographs illustrating different steps of the method described. (a)** Etiolated *Cucumis sativus* seedlings 21 days after germination showing extensive root development before nematode inoculation. **(b)** Close-up of *Meloidogyne javanica* infected *C. sativus* roots showing the galls induced by this nematode and the egg masses deposited by the females outside the root (black arrowheads). **(c)** Close-up of a gall containing one female laying an egg mass with amber color in an optimum stage for hatching to obtain vigorous J2s. Hatched J2s around the egg mass are marked by white arrowheads. **(d)** Close-up of an unviable dark brown egg mass containing juveniles which are often dead. The dead J2s are easily observed around the egg mass and gall (yellow arrowheads). Scale bars: 1 cm **(a,b)** and 200 μm **(c,d)**.

Fifty *C. sativus* (L.) cv. Hoffmanns Giganta seeds (Buzzy Seeds, Catalog Number: 02186) were surface-sterilized with 40 mL of undiluted commercial bleach (35 gr/L) for 45 min and subsequently washed five times with sterile distilled water under a laminar flow hood. Ten Petri dishes (14 cm diameter) containing modified Gamborg B5 solid media (see Supplemental Material) supplemented with 3% sucrose were used to sow five seeds/plate with sterile tweezers (**Figure 1**). Plates were sealed with one layer of Parafilm^®^ first, then with Micropore^®^ tape and finally covered with aluminum foil to favor the development of the root system in darkness and avoid contamination, as it should be a long-lasting axenic culture. After 2 days of stratification at 4°C, the plates were transferred to a dark growth chamber at 26°C for 21 days (**Figures 1 and 2a**). Just before inoculation with J2s, the etiolated aerial parts of the cucumber seedlings were removed to promote further root growth.

### Nematode Inoculation

Cucumber plates used to collect the egg masses (**Figure 2a**) had to be carefully checked under a stereo-microscope to detect any visible contamination. Egg masses selected for hatching should have an amber color (**Figures 2b,c**) when they are around 2 months old. Darker brown egg masses (**Figure 2d**) should be avoided as they are old and will produce a lower number of less vigorous nematodes. It is recommended to observe some hatched J2 moving around the egg masses as this normally indicates that J2 are viable (**Figure 2c**). When egg masses are old, several dead juveniles are frequently found around them (**Figure 2d**). Four days before inoculation, 50 sterile egg masses (from one or several previous plates of cucumber plants 2 months after inoculation; **Figure 1**) were placed in a hatching jar in a sterile cell strainer (with a 70 μm nylon mesh) inside a beaker filled with 5 mL of sterile tap water (see Supplemental Material). The mesh retained the egg masses while the hatched J2s moved through the sieve and subsequently sank to the bottom of the beaker. Hatching took place in the dark at 26°C for 4 days, and 1 mL with the freshly hatched J2s was used to inoculate the cucumber plates every 23 days (**Figure 1**). In general, one Petri dish is enough to provide the 50 egg masses needed to inoculate the 10 new cucumber plates; the remaining nine infected cucumber plates in each batch can be used for hatching J2s for *in vitro* experiments (**Figure 1**). Plates were inoculated with 1 ml from the hatching jar, comprising approximately 1000 J2 (**Figure 2a**, Supplemental Material). After J2 inoculation, the plates were double sealed, covered with aluminum foil and placed back into the growth chamber for 2 months (**Figure 1**) till lifecycle completion, when new egg masses were produced. These egg masses can be used again to obtain more juveniles for new cucumber seedling inoculation (**Figure 1**). Instead of freshly hatched J2s, egg masses from the infected cucumber seedlings could also be used to inoculate new plates. In this case, from three to five egg masses per plate were placed on the agar medium near the roots with the help of sterile tweezers. However, this procedure can cause many asynchronous infections, as juveniles hatch gradually from the eggs. It is also important to point that if required, several amplification rounds can be performed from a plate infected with a single egg mass from a single female. This can be crucial in some experiments where clonal nematodes are required to reduce variability. Once the culture is stablished, we recommend to use the plates from this clonal amplification of females for further experiments and amplifications. It is advisable to settle a new stock of cucumber plates every 21 days, so that several different stock plates at different stages are generated (**Figure 1**).

## Results and Discussion

**FIGURE 3:**
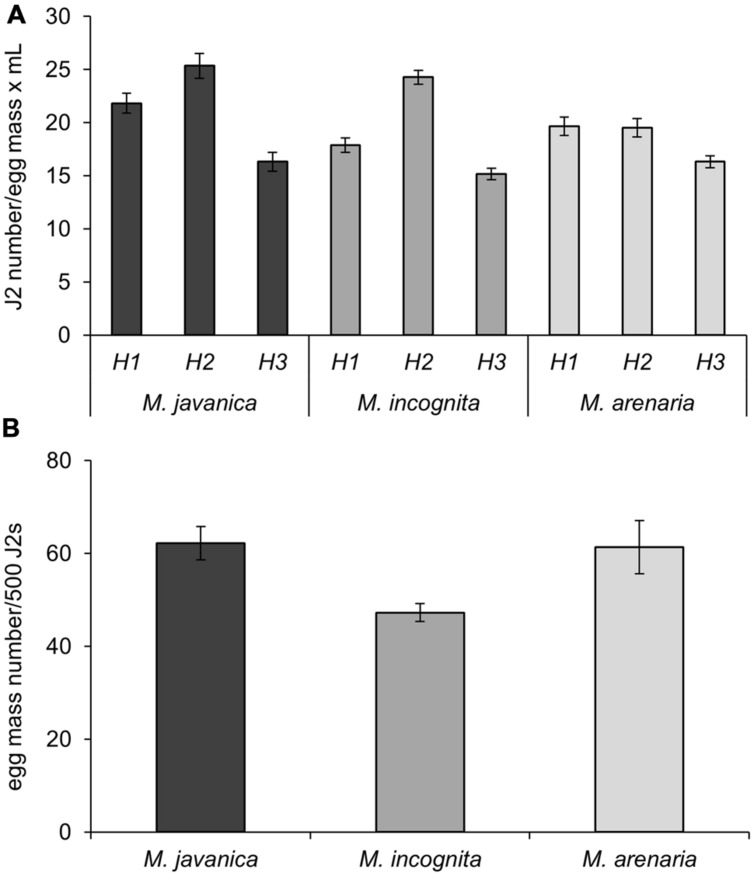
**Nematode production. (A)** Number of J2s from *M. javanica*, *M. incognita*, and *M. arenaria* per egg mass obtained after hatching in sterile tap water. H1, H2, and H3 correspond to three consecutive rounds of hatching for 4 days each at 26°C carried out in 5 mL of sterile tap water in the same hatching jar. **(B)** Number of egg masses obtained from a plate with five seedlings of *C. sativus* grown for 21 days inoculated with 500 J2. Three different species of root-knot nematodes were assessed as indicated.

We determined the hatching ability of egg masses produced following the protocol described above for three different RKN species: *Mj*, *Mi*, and *Ma*. Fifty egg masses (2-month-old) obtained from a single female clonal culture were collected for each species and incubated for 4 days in 5 mL of sterile tap water (see Supplemental Material) in a hatching jar. The number of J2 was assessed by counting under the stereo-microscope in three aliquots of 30 μl each. The average J2 number among the three replicates was considered as a good estimation for the total number of hatched J2s. *Mj* egg masses yielded the highest number of J2s per egg mass and per mL (21.8 J2s/em⋅mL; **Table 1**). A slightly smaller number was obtained for *Ma* (19.7 J2s/em⋅mL) and *M*i (17.9 J2s/em⋅mL; **Figure 3A**; **Table 1**). After the first hatching was collected, a new volume of 5 mL of sterile tap water was added to the hatching jar to favor a second hatching round from the same masses for another 4 days. The number of juveniles obtained in this second round was higher than in the first hatching for *Mj* and *Mi* (**Figure 3A**; **Table 1**; 25.3 and 24.3 J2s/em⋅mL, respectively), while the number was maintained for *Ma* (**Figure 3A**; **Table 1**). In a third hatching round, under the same conditions, the number of juveniles decreased in comparison with the first and second rounds for all species (**Figure 3A**; **Table 1**). Each hatching jar contained 50 egg masses in 5 mL of sterile distilled water, and in each hatching round an average of 5290, 4777, and 4625 J2s of *Mj*, *Mi*, and *Ma*, respectively (**Figure 3A**; **Table 1**) were obtained that could be used for inoculation of plants grown *in vitro*. The sum of all three hatchings from the 50 egg masses of each species yielded a total number of 15869, 14330, and 13875 juveniles from *Mj*, *Mi*, and *Ma*, respectively (**Figure 3A**; **Table 1**). As the nematodes came from a monoxenic culture, there was no need for chemical sterilization of juveniles. These treatments reduce their vigor and viability and usually results in a high variation in the infection ability of the J2s, ranging from very inefficient infection to wounding effects when too many nematodes tended to penetrate the same root. In contrast, J2s from the described aseptic culture were in the optimum infectivity state that did not vary much in different batches. This allowed the use of a reduced nematode inoculum (10 nematodes per plant) to avoid undesired root damage (Cabrera et al., 2014, 2015). Another advantage of the method herein described is that it allows for three independent biological replicates by infecting plants 4 days apart with J2 hatched from the same egg mass pool from three independent hatchings. This method also contributes to the homogeneity of the infection efficiency, reducing variability among experiments. All hatching data presented here are the average of more than 20 amplification rounds (*n* = 45 for *Mj*; *n* = 20 for *Mi* and *Ma*) performed during the last 5 years (10 years after the initial inoculation). It is important to point that the amplification ability of the population may have changed since the first set of infections took place 15 years ago.

**Table 1:** Hatching rate and reproduction parameters for three *Meloidogyne* spp. in cucumber root cultures.

Nematode species	*Meloidogyne javanica*				*Meloidogyne incognita*				*Meloidogyne arenaria*			
Hatching round	1st	2nd	3rd		1st	2nd	3rd		1st	2nd	3rd	
J2 number /egg mass × mL	21.8	25.3	16.3		17.9	24.3	15.2		19.7	19.5	16.3	
J2 number/50 egg masses × 5 mL	5453.7	6336.5	4078.9		4467.8	6067.3	3795.3		4916.7	4880.6	4077.8	
Average (J2/50 egg masses) in each hatching round				5289.7				4776.8				4625.0
Total in the three hatching rounds				15869.1				14330.4				13875.0
J2 number/mL^∗^	1090.7	1267.3	815.8		893.6	1213.5	759.1		983.3	976.1	815.6	
Average				1057.9				955.4				925.0
Total J2 in the three hatches per species				3173.8				2866.1				2775.0
No. of egg masses/500 J2s inoculated in each plate				62.2				47.3				61.4
No. of egg masses/1 mL of inoculum per plate	135.7	157.7	101.5		84.5	114.7	71.8		120.7	119.8	100.1	
Average per species				131.6				90.3				113.5
No. of J2s/total No. of egg masses per plate				41776.3				25888.9				31497.2
Pf/Pi (ratio between final and initial J2 population)				39.5				27.1				34.1

^∗^*Used as inoculum for one cucumber plate.*

Juveniles from *Mj, Mi*, and *Ma* obtained from monoxenic cultures were used to inoculate plates containing five etiolated cucumber seedlings 23 days after sowing. Two months after inoculation, the number of egg masses developed in each plate was counted under a stereo-microscope and, subsequently, the “No. of egg masses per 500 J2s of the initial inoculum” was calculated (**Table 1**; **Figure 3B**). Each plate was inoculated with 1 mL of sterile tap water containing J2s from the hatching jar from any of the hatching rounds (see **Table 1** for nematode numbers). Thus, the average number of J2 in the inoculum was 1058, 955, and 925 for *Mj*, *Mi*, and *Ma*, correspondingly (**Table 1**). With this inoculum, it was possible to obtain an average of 132, 90, and 113 egg masses for each species in each plate (**Table 1**). *Mj* and *Ma* juveniles seemed to be the species with the greatest capacity to reproduce *in vitro* in cucumber roots as 62 and 61 egg masses were obtained in each plate per 500 J2 from the initial inoculum (**Table 1**; **Figure 3B**). The number of egg masses obtained from *Mi* was slightly smaller; 47 egg masses per plate inoculated with 500 J2 (**Figure 3B**). All three species reproduced efficiently in cucumber roots, however, *Mj* showed the best hatching and reproduction parameters in our system (**Figure 3**; **Table 1**). The number of juveniles from any of the three species could be increased ten-fold from the initial inoculum when 10 plates with five cucumber seedlings were used. Depending on the number of hatched J2 obtained per each egg mass, it is possible to obtain a total number of 41776, 25888, and 31497 J2s for *Mj*, *Mi*, and *Ma* (**Table 1**), respectively, from all the egg masses produced in a single cucumber plate. Data from tomato roots transformed with *A. rhizogenes in vitro* infected with an aggressive *M. hapla* population indicated a production of 20000 nematodes per plate after 8 weeks of growth (Mitkowski and Abawi, 2002). Regardless of the different *Meloidogyne* sp. we obtained for all three species a higher amplification rate in a similar period. Moreover, for *Mj* it almost doubled that of *M. hapla* in transformed roots. Comparison with amplification methods in soil are precluded, as the plant growing conditions also influence nematode reproduction.

The amplification ratio from the initial J2 population used for inoculation (Pf/Pi) was 39.5 (*Mj*), 27.1 (*Mi)*, and 34.1 (*Ma)* (**Table 1**). This is in the range obtained with *A. rhizogenes* transformed roots from bindweed, bean, carrot and tropical tomato for *M. javanica*, but lower than the amplification obtained in potato and tomato (*Solanum lycopersicum* Mill. cv. South Australian Early Dwarf Red) transgenic roots (Pf/Pi = 83 and 161, respectively; Verdejo et al., 1988). Although, this last method based on *in vitro* infected transgenic roots is sufficient to amplify *Meloidogyne* spp. in monoxenic cultures, the method based on cucumber roots is simpler, as it does not require root transformation. When cucumber root plates get contaminated, a new batch of seeds can be easily germinated. On the other hand, when hairy root systems are used, extensive contamination of transformed roots plates may require a new transformation event.

Here, we report the use of cucumber as a suitable host for *Meloidogyne* spp. maintenance in monoxenic cultures. We have been able to amplify different *Meloidogyne* spp. over 15 years routinely in the laboratory. Cucumber can be easily cultivated *in vitro* as its seedlings develop a dense root system within a short time. Thus, amplification of *Mj*, *Mi*, and *Ma* populations of at least 39.5, 27.1, and 34.1 times, respectively, was achieved from the initial J2 inoculum. The differences in the amplification ratio among the different species are probably caused by diverse virulence of the nematode populations used (Semblat et al., 2000).

Finally, our system is suitable as it is low-cost and no time-consuming as well as it requires basic laboratory equipment. In addition, it is easily restored in case of *in vitro* culture contamination or an accidental heat shock or other problems caused by growth chamber failure. Our main proof of the suitability of the method is that a population of *Mj* started from a single female and was maintained in a cucumber monoxenic culture in our laboratory after following the protocol presented here for more than 15 consecutive years.

## Author Contributions

All authors substantially contributed to the acquisition (FEDM, RO, MB, JC), analysis (FEDM, JC, CE) or interpretation (FEDM, RO, MB, JC, CE, CF) of the data presented in this manuscript. All authors drafted and approved the manuscript. All authors are accountable for all aspects of the work and ensure that questions related to the accuracy or integrity of any part of the work are appropriately investigated and resolved.

## Conflict of Interest Statement

The authors declare that the research was conducted in the absence of any commercial or financial relationships that could be construed as a potential conflict of interest.
