# Supplementary material for: Long-Term In Vitro System for Maintenance and Amplification of Root-Knot Nematodes in Cucumis sativus Roots
Source: Front Plant Sci. 2016 Feb 22;7:124. doi: 10.3389/fpls.2016.00124 (PMC4761865; doi:10.3389/fpls.2016.00124)
Supplement: Supplementary file 1 [file Presentation_1.PDF]

SUPPLEMENTAL MATERIAL AND METHODS: DETAILED PROTOCOL

A) Biological Materials and Reagents:

- *Cucumis sativus* L. cv. Hoffmanns Giganta seeds, also known as cv. Hoffmanns Johanna, (Buzzy Seeds, Catalog Number: 02186).
- Root-knot nematodes, *Meloidogyne* spp.: *M. javanica* (*Mj*), *M. incognita* (*Mi*) and *M. arenaria* (*Ma*).
- Gamborg® B5 Basal salt mixture including vitamins (Duchefa®, Catalog Number: G0210.0050).
- Sucrose (Duchefa, Catalog Number: S0809.1000).
- Daishin agar (Duchefa, Catalog Number: D1004.1000).
- Potassium hydroxide (KOH; Duchefa, Catalog Number: 000517).
- Commercial bleach (35 gr. of active chlorine per liter).
- Sterile distilled water.
- Sterile tap water.

B) Equipment:

- Parafilm®.
- Micropore® surgical tape.
- 1.5 ml Eppendorf® tubes.
- 140 mm Ø Petri® dishes.
- 50 ml conical tubes.
- Tweezers.
- Glass bead sterilizer.
- Aluminum foil.
- Scalpel handle and scalpel blades.
- Scissors.
- Growth chamber at 26ºC (no light needed).
- Laminar flow hood cabinet.
- Sterile cell strainer (70 µm nylon mesh) (BD Falcon™, Catalog Number: 352350).
- 50 mL beaker.
- 300-400 mL glass jar with hermetic lid.
- Stereo-microscope.

C) Modified Gamborg® B5 culture medium:

- 30 g/L Sucrose.
- 3.05 g/L Gamborg® B5 Basal salt mixture including vitamins.
- 8 g/L Daishin Agar.
- Adjust pH to 6.4 with 1M KOH.

D) Procedure:

Note: Use gloves under a laminar flow cabinet throughout all procedures.

1. Preparation of plates with growth medium:

- Prepare 1 L of modified Gamborg® B5 medium as indicated and autoclave (121ºC for 20 min at 1 atm).

- Pour media into 140mm Ø Petri® dishes (around 100ml/plate) and let them solidify.

Note 1: Plates can be Parafilm®–sealed and stored at 4ºC if they are not going to be used immediately. Do not allow accumulation of liquid on the surface, as it will promote future contaminations.

2. *Cucumis sativus* seeds - sterilization and sowing:

- Surface-sterilize *C. sativa* seeds with 40 mL undiluted commercial bleach (35 gr/L) for 45 minutes in constant agitation in a 50 mL conical tube. It is recommended to use around 60 seeds per tube.

Note 2: From this step onwards, the protocol should be carried out under sterile conditions in a laminar flow cabinet.

- Discard the bleach and rinse the seeds five or six times with sterile distilled water (approximately 30mL per rinse) in a 50mL conical tube.

- Immediately after the last washing step, place five seeds of *C. sativus* with the help of sterile tweezers in the previously prepared modified Gamborg® B5 media plates.

Note 3: Tweezers should be previously sterilized. For this purpose, we use a glass bead sterilizer, which allows quick re-sterilization of tweezers when necessary.

- Seal the plates with one layer of Parafilm® first and then with Micropore® tape. Cover them with aluminum foil to favor the development of the root system in darkness.

Note 4: Long incubating periods at 26ºC (two months or longer) will make Parafilm® tear, favoring plate contamination. The extra Micropore® layer helps to avoid any contamination.

- Keep the plates for two days at 4 °C for seed stratification, thereby promoting the synchronous germination of all the seeds.

- Transfer the plates to a growth chamber in darkness at 26°C for 21 days. Humidity control is not strictly necessary.

Note 5: In order to guarantee the stock of the nematode population plates, we strongly recommend maintaining at least 10 plates of inoculated *C. sativus* roots per nematode species and making a new batch every 21 days.

3. *C. sativus* plate inoculation

- Prepare a hatching jar by placing a cell strainer in a 50ml beaker. After sealing the beaker with aluminum foil, insert it into a bigger container (e.g. a glass container with a lid; see images below) and autoclave it under the same conditions as those described for the media.

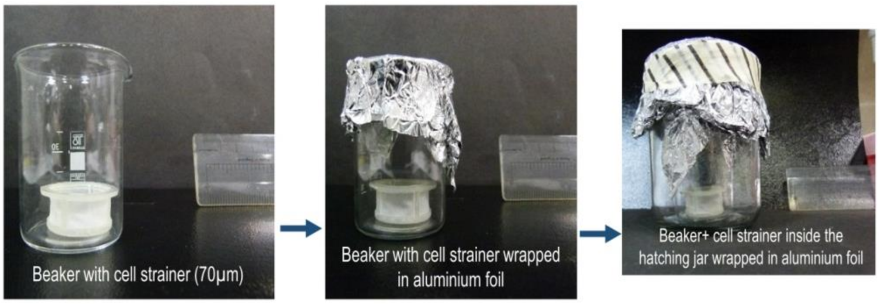

- Add 5 ml of sterile tap water (tap water is important, as empirically we observed that the process is more effective than with distilled water) to the beaker, under a laminar flow cabinet. This volume is sufficient to cover the nylon mesh of the cell strainer.

- Collect 50 egg masses inside the laminar flow cabinet, with the help of sterile tweezers, from previously obtained monoaxenic plates and place them in the cell strainer.

Note 6: Before collecting the egg masses, it is advisable to check the sealed cucumber plates under a stereo-microscope for the presence of any contaminating microorganism (i.e., yeast or fungi) and to locate with a marker those egg masses with the appropriate color. Hence, egg masses selected for hatching should be those with an amber color, usually two months old (see image below).

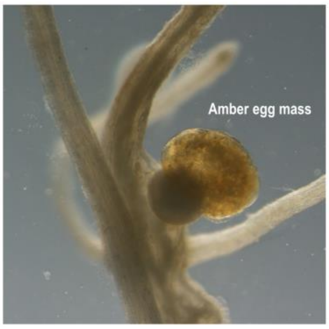

If they have turned darker brown, this is an indication that they are old and a lower number and/or less vigorous nematodes will hatch than from amber-colored egg masses. It is also recommended to observe some hatched J2 moving around the egg masses (see Figure 2).

- Assemble the hatching jar again and close it tightly to preserve the sterility.
- Place the hatching jar in a dark growth chamber at 26 °C for 4 days.

Note 7: The hatching step must be performed four days before the planned inoculation time.

- To promote root growth and to facilitate the subsequent handling of the egg masses, cotyledons and elongated hypocotyls are removed with a sterile scalpel and tweezers (see image below). Removal of aerial parts and plant inoculation are recommended at the same time to avoid extra manipulation of the plates, and hence to decrease the chance of contamination.

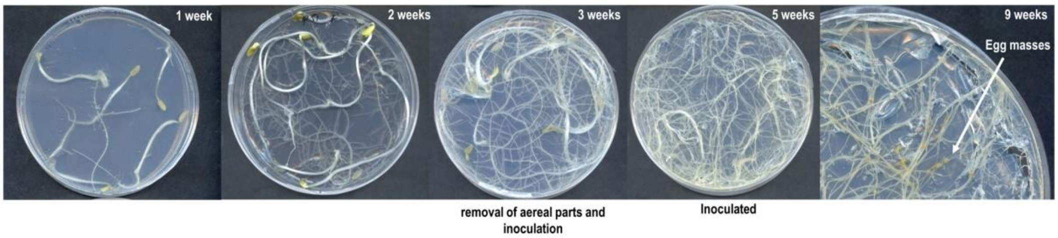

Note 8: When removing the aerial part of germinated seeds replace the sterile scalpel and re-sterilize tweezers every two plates to minimize contamination. Thus the use of a glass bead sterilizer is very convenient for fast re-sterilization of the instrument.

- Nematode inoculation should be carried out in cucumber seedlings with fully developed root systems, approximately 21-day-old seedlings as shown.
- Shake the hatching jar briefly to get a homogeneous solution before taking the aliquots for J2 counting.
- Assess the number of hatched J2/mL under a stereo-microscope by counting the number of J2 in three independent 30 µl drops from the hatching jar, estimating the average among the three measurements.

- For efficient root infection (see Results; Figure 1), inoculate each plate with around 1000 juveniles (J2), which usually corresponds to 1 mL of the hatching suspension. Add 1 mL of the sterile tap water with the previously estimated J2 from the hatching jar with an automatic pipette to the surface of each cucumber plate, distributing the volume across the whole plate. **Note 9**: Instead of freshly hatched J2s, egg masses from previously infected cucumber seedlings could also be used to inoculate new plates. In this case, three to five egg masses/plate are placed on the agar medium with the help of sterile tweezers. However, keep in mind that this procedure will make many asynchronous infections, as juveniles hatch gradually from eggs.

- Seal the plates again with Parafilm® and Micropore® tape to prevent contamination. Wrap all of them with aluminum foil.

- Place the plates back in the growth chamber at 26 °C in darkness for about two months. This is long enough for the root-knot nematodes to complete their lifecycle in cucumber roots and to produce new egg masses that can be used to hatch juveniles to inoculate new cucumber seedlings or to perform experiments using other plant species.

Note 10: It is important that the growth chamber is not completely full of plates to facilitate oxygen diffusion to the plates. As previously indicated, it is advisable to settle a new stock of cucumber plates every 21 days. A population newly planted (cucumber seeds one week old), another population about to be inoculated (cucumber seeds three weeks old) and two more about to be hatched (cucumber seeds 8 to 12 weeks old) are recommended to be maintained simultaneously as stocks. Following this schedule, we have been able to maintain *in vitro* cultures for *Meloidogyne* spp. for 15 years since they were initially isolated from soil-grown plants.
